# Impact of Cognitive Reserve and Structural Connectivity on Cognitive Performance in Multiple Sclerosis

**DOI:** 10.3389/fneur.2020.581700

**Published:** 2020-10-30

**Authors:** Elisabet Lopez-Soley, Elisabeth Solana, Eloy Martínez-Heras, Magi Andorra, Joaquim Radua, Albert Prats-Uribe, Carmen Montejo, Nuria Sola-Valls, Maria Sepulveda, Irene Pulido-Valdeolivas, Yolanda Blanco, Elena H. Martinez-Lapiscina, Albert Saiz, Sara Llufriu

**Affiliations:** ^1^Laboratory of Advanced Imaging in Neuroimmunological Diseases, Center of Neuroimmunology, Institut d'Investigacions Biomediques August Pi i Sunyer (IDIBAPS), Hospital Clinic Barcelona, Universitat de Barcelona, Barcelona, Spain; ^2^Imaging of Mood- and Anxiety-Related Disorders (IMARD) Group, Mental Health Research Networking Center (CIBERSAM), Institut d'Investigacions Biomèdiques August Pi i Sunyer (IDIBAPS), Barcelona, Spain; ^3^Department of Psychosis Studies, King's College London, Institute of Psychiatry, Psychology and Neuroscience, London, United Kingdom; ^4^Department of Clinical Neuroscience, Centre for Psychiatric Research and Education, Karolinska Institutet, Solna, Sweden; ^5^Centre for Statistics in Medicine, Botnar Research Centre, Nuffiel Department of Orthopeadics, rheumatology and musculoskeletal sciences (NDORMS), University of Oxford, Oxford, United Kingdom

**Keywords:** cognitive reserve, structural connectivity, graph theory, cognition, multiple sclerosis

## Abstract

**Background:** Cognitive reserve (CR) could attenuate the impact of the brain burden on the cognition in people with multiple sclerosis (PwMS).

**Objective:** To explore the relationship between CR and structural brain connectivity and investigate their role on cognition in PwMS cognitively impaired (PwMS-CI) and cognitively preserved (PwMS-CP).

**Methods:** In this study, 181 PwMS (71% female; 42.9 ± 10.0 years) were evaluated using the Cognitive Reserve Questionnaire (CRQ), Brief Repeatable Battery of Neuropsychological tests, and MRI. Brain lesion and gray matter volumes were quantified, as was the structural network connectivity. Patients were classified as PwMS-CI (*z* scores = −1.5 SD in at least two tests) or PwMS-CP. Linear and multiple regression analyses were run to evaluate the association of CRQ and structural connectivity with cognition in each group. Hedges's effect size was used to compute the strength of associations.

**Results:** We found a very low association between CRQ scores and connectivity metrics in PwMS-CP, while in PwMS-CI, this relation was low to moderate. The multiple regression model, adjusted for age, gender, mood, lesion volume, and graph metrics (local and global efficiency, and transitivity), indicated that the CRQ (β = 0.26, 95% CI: 0.17–0.35) was associated with cognition (adj *R*^2^ = 0.34) in PwMS-CP (55%). In PwMS-CI, CRQ (β = 0.18, 95% CI: 0.07–0.29), age, and network global efficiency were independently associated with cognition (adj *R*^2^ = 0.55). The age- and gender-adjusted association between CRQ score and global efficiency on having an impaired cognitive status was −0.338 (OR: 0.71, *p* = 0.036) and −0.531 (OR: 0.59, *p* = 0.002), respectively.

**Conclusions:** CR seems to have a marginally significant effect on brain structural connectivity, observed in patients with more severe clinical impairment. It protects PwMS from cognitive decline regardless of their cognitive status, yet once cognitive impairment has set in, brain damage and aging are also influencing cognitive performance.

## Introduction

Cognitive impairment (CI) has been reported in 40–70% of people with multiple sclerosis (PwMS) (1) and it has a negative impact on their quality of life (2). It is associated with the combined effect of both white matter (WM) and gray matter (GM) damage (3). However, magnetic resonance imaging (MRI) metrics like lesion volume (Lv) or GM volume (GMv) only partially explain the cognitive changes of PwMS. Non-conventional MRI techniques, such as diffusion-weighted imaging (DWI) or functional MRI (fMRI), can be used to further explore structural and functional brain connectivity and its associations with CI (4, 5). Moreover, through theoretical graph analysis, it has been suggested that disrupting the optimal balance between local integration and global segregation of network components might hamper information flow, exerting a negative impact on cognition (5, 6).

Some individuals better maintain their cognitive performance despite the presence of substantial brain damage. This clinico-pathological dissociation (7) indicates that certain factors protect against cognitive decline, such as the cognitive reserve (CR), understood to be lifelong intellectual enrichment that attenuates the negative effect of MS disease burden on neuropsychological activity (8, 9). Previous studies in MS and other neurodegenerative diseases, such as Alzheimer's disease, suggested that patients with higher CR displayed better cognitive function regardless of having similar brain damage (7, 10, 11). Indeed, CR seems to preserve brain network functional connectivity counterbalancing the impact of the disruption of WM tracts due to lesions in MS on cognition (12). However, this protective role of the CR diminishes over the MS disease course as the brain burden becomes stronger (13, 14). Before the appearance of CI, the brain probably employs adaptive and compensatory mechanisms, undergoing structural and functional reorganization in response to the pathological changes caused by MS (5, 15). However, the accumulation of brain damage can lead to network dysfunction that may contribute significantly to the development of CI in PwMS (16). As far as we know, the relationship between CR and structural brain connectivity remains unexplored.

We hypothesized that individuals with higher CR would exhibit higher structural connectivity and, consequently, better cognitive performance. Also, the influence of CR on cognitive performance in PwMS may be distinct before and after the emergence of CI. Therefore, we aimed to understand the association between CR and structural connectivity integrity and their impact on cognition in PwMS. For this, we analyzed their role in patients with different cognitive status, thus in PwMS cognitively impaired (PwMS-CI) and in those who remained cognitively preserved (PwMS-CP).

## Materials and Methods

### Participants

A cohort of 181 PwMS (aged 18–65 years) who fulfilled the 2010 McDonald criteria (17) was consecutively selected at the MS Unit of the Hospital Clinic of Barcelona. To be included, patients had to be free from relapses in the last 30 days and have no significant neurological or psychiatric condition that could interfere with cognitive functioning. In this cross-sectional study, patients were evaluated using clinical and cognitive scales, and they underwent an MRI scan. We collected data regarding MS type, disease duration, current treatment, and global disability, the latter measured using the Expanded Disability Status Scale (EDSS) (18). In addition, a global score of depression and anxiety symptoms was obtained for the patients using the Hospital Anxiety and Depression Scale (HADS) (19). The Ethics Committee at the Hospital Clinic of Barcelona approved the study and all the participants signed an informed consent form prior to their enrollment on the study.

### Neuropsychological Assessment

Cognition was assessed using the Brief Repeatable Battery of Neuropsychological tests (BRB-N) (20). This battery includes different tests assessing cognitive domains as follows: (1) verbal learning and memory: Selective Reminding Test (SRT, with two subtests: consistent long-term retrieval as an indicator of consolidation, and delayed retrieval); (2) visuospatial learning and memory: 10/36 Spatial Recall Test (SPART, with two subtests: immediate retrieval and for delayed retrieval); (3) attention, working memory, and information processing speed: Symbol Digit Modalities Test (SDMT) and Paced Auditory Serial Addition Test (PASAT) 3 s per digit version; and (4) verbal fluency and cognitive flexibility: Word List Generation (WLG).

We first calculated *z* scores for all BRB-N tests, using demographically adjusted (age and education) regression models according to the normative data published in the Spanish population (21), classifying patients as PwMS-CI or PwMS-CP. Patients were classified as PwMS-CI if performance was below *z* = −1.5 standard deviations (SD) of the norm in at least two cognitive tests of the same or different cognitive domain. In addition, raw values were transformed into *z* scores (zBRB) by subtracting the mean and dividing by the SD of the whole sample in order to obtain a mean score of cognitive performance, avoiding the educational effect related to CR and the aging effect on cognition.

### Assessment of CR

CR was assessed using the Cognitive Reserve Questionnaire (CRQ) (22), a standardized scale in which higher scores represent higher levels of CR (maximum 25 points). This test is composed of eight items that measure different intellectual enrichment factors, including the individual's education, their parent's education, training courses, occupation, musical training, language studies, reading activity, and intellectual games in which they have participated during their adult lifetime. Items do not contemplate a specific period, thus addressing experiences throughout life (23). This questionnaire was administered by an experienced neuropsychologist before the cognitive assessment. The CRQ has been previously applied to both healthy elderly and diseased populations (24, 25).

### Magnetic Resonance Images

#### MRI Acquisition

MR images were acquired on a 3-Tesla Magnetom Trio (SIEMENS, Erlanger, Germany) scanner using a 32-channel phased-array head coil. The protocol applied involved a 3D-Magnetization Prepared Rapid Acquisition Gradient Echo (MPRAGE), 3D-T2 fluid-attenuated inversion recovery (FLAIR), and DWI sequences (see Supplementary Material for a detailed description of the sequences).

#### Structural MRI Processing for Volumetric Analysis

WM lesions were defined semi-automatically on the 3D-MPRAGE sequence using the Jim7 software (http://www.xinapse.com/j-im-7-software/). To improve MS lesion identification, the co-registered 3D-FLAIR image was used as a reference. Thereafter, lesion in-painting was applied to the 3D-MPRAGE image to enhance segmentation and registration in PwMS (26). The FSL and SIENAX tools (27) were used to obtain the normalized Lv and GMv (nLv and nGMv).

#### Whole Brain Structural Connectivity Reconstruction

Cortical parcellation was performed with the Mindboggle software (28) using a cortical labeling parcellation scheme from FreeSurfer (https://surfer.nmr.mgh.harvard.edu/) that is based on the Desikan-Killiany atlas (29). Subcortical GM structures were segmented by applying the FIRST tool (fsl.fmrib.ox.ac.uk/fsl/fslwiki/FIRST). Thirty-one cortical regions and seven subcortical GM structures per hemisphere were used as nodes of the network.

DWI processing was performed as described previously (5, 30). High Angular Resolution Diffusion Imaging (HARDI) images were denoised and corrected for geometric distortions and head motion (31). The structural connectome was obtained using multi-tissue constrained spherical deconvolution-based tractography, applying the second-order integration over fiber orientation distributions and an anatomically constrained tractography framework (32), available in the MRtrix3 software package (http://www.mrtrix.org/). WM and lesion masks were registered to the undistorted HARDI images by applying boundary-based registration (33). Fiber tracking required a seeding mask that corresponded to the normal-appearing WM and MS lesions, thereby avoiding premature cessation of the reconstruction in areas with a more complex structural architecture and with low fractional anisotropy (FA) (30). Anatomical exclusion criteria were applied to minimize the number of anatomically aberrant connections originated from the tractography procedure (30). Finally, the total 76 segmented cortical and subcortical regions were used to define the nodes of the network, and matrices were generated to represent the mean FA values of the connections.

#### Network Analysis

**Figure 1:**
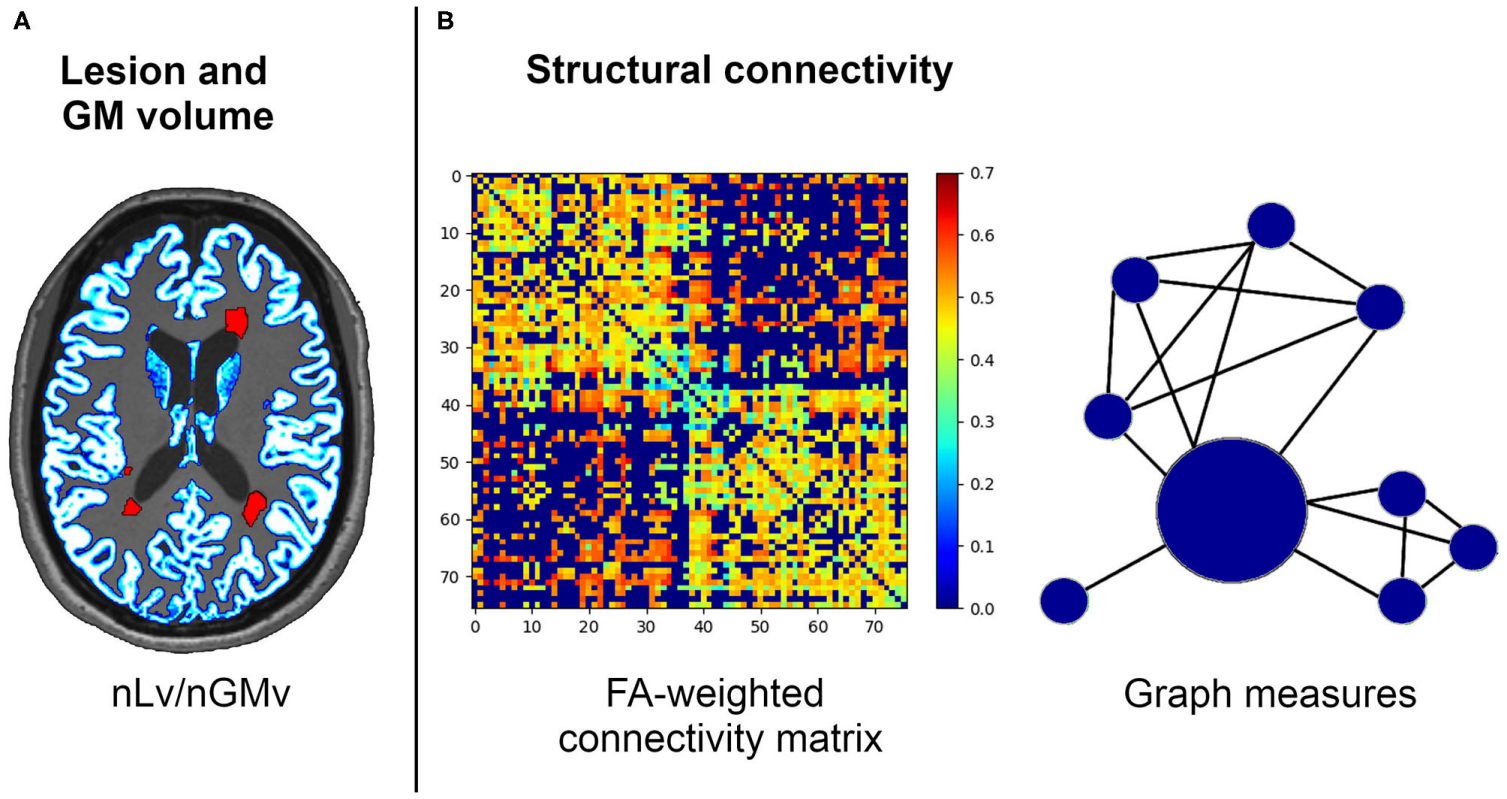
Structural connectivity framework for the neuroimaging processing of the volumetric **(A)** and network **(B)** analysis. FA, fractional anisotropy; nGMv, normalized gray matter volume; nLv, normalized lesion volume.

Graph theory metrics were computed using the Brain Connectivity Toolbox (https://sites.google.com/site/bctnet). Graph metrics were analyzed to express the global connectivity properties of the network, including the nodal strength (the sum of weights connected to the node); measures of segregation, such as the local efficiency (the average of the inverse of the shortest path length in the network computed on node neighborhoods), the clustering coefficient (the fraction of a node's neighbors that are neighbors of each other), and transitivity (the ratio of triangles to triplets in the network); integration, as measured through the global efficiency (the average inverse shortest path length in the whole network); and brain resilience, reflected by assortativity (a correlation coefficient of the degrees of separation of all the nodes at two opposite ends of a link) (34). A representative image of the MRI metrics used in this study is presented in Figure 1.

### Statistical Analysis

All demographic, clinical, neuropsychological, MRI markers of brain burden, and connectivity values were described through the mean and SD, and by the absolute numbers and the proportions for quantitative and qualitative data, respectively. The normality of continuous data was checked using histograms and appropriate statistical methods as the Shapiro–Wilks test. We compared the aforementioned characteristics between PwMS-CI and PwMS-CP patients using a Chi-squared test, a Wilcoxon–Mann–Whitney *U*-test or a Student's *t*-test, depending on the data distribution.

Age- and gender-adjusted linear regressions were done to analyze the associations between CRQ and cognition, between structural connectivity and cognition, and between CRQ and structural connectivity on the entire cohort and in each group of PwMS separately. To understand the role of CR and structural connectivity on cognitive performance in the context of other demographic and MS-related factors, we fitted a multiple regression model that included relevant demographic and clinical variables. Variables were standardized using the mean and SD: CRQ score, age, gender, EDSS, HADS score, nLv, nGMv, and graph measures of segregation, integration, and brain resilience (nodal strength, global and local efficiency, clustering coefficient, transitivity, and assortativity). The Akaike Information Criterion (AIC) was then used to select the variables that best fit a model based on the whole cohort. As the main objective of the study was to determine the influence of CR and structural connectivity on cognitive performance in patients with different cognitive status, we applied the same multiple regression model separately in PwMS-CI and PwMS-CP, with the variables selected from the AIC. We computed the strength of the associations using Hedges' *g* effect size.

Furthermore, an age- and gender-adjusted logistic regression analysis was used to estimate the odds ratio (OR) of having an impaired cognitive performance associated with the increase per unit of the CR and MRI connectivity metrics associated to cognition in the multiple regression model.

Statistical analyses were performed with R statistical software (version 3.6.0, www.R-project.org), setting the level of significance at *p* < 0.05 and correcting multiple comparisons for the false discovery rate (FDR).

## Results

This study was carried out on a population of 181 PwMS who were mostly female (71%), middle-aged adults (42.9 ± 10.0 years), and who had a median CRQ score of 16 (interquartile range, IQR: 12–24). In the cohort, 81 patients (45%) were classified as PwMS-CI, and the remaining 100 patients (55%) were considered PwMS-CP. The group of PwMS-CI more frequently presented with a secondary progressive phenotype of the disease, with lower CRQ scores and with higher EDSS scores. They also presented worse volumetric and connectivity measures in images than PwMS-CP, although assortativity was no different (see Table 1 for further details).

**Table 1:** Demographic, clinical, and MRI data of the study population.

	**Entire cohort (*n* = 181)**	**Cognitive status groups**		
		**PwMS-CP (*n* = 100)**	**PwMS-CI (*n* = 81)**	***p*-value**
**Demographic data**				
Female, *n* (%)	128 (71)	76 (76)	52 (64)	0.116^a^
Age (years)	42.9 (10.1)	41.4 (9.0)	44.7 (11.0)	0.027^c^
CRQ score median [IQR]	16 (12–19)	17 (11–18)	15 (11–18)	0.014^b^
Education level				
Basic (0–8 years)	11 (6)	6 (6)	5 (6)	0.036^a^
Primary (9–12 years)	76 (42)	40 (40)	36 (45)	
Secondary (13–16 years)	58 (32)	40 (40)	18 (22)	
Higher (>17 years)	36 (20)	14 (14)	22 (27)	
Right handed	158 (87)	88(88)	70 (86)	0.523^a^
**Clinical data**				
Type of MS, *n* (%)				
RRMS	166 (92)	96 (96)	70 (86)	0.040^b^
SPMS	15 (8)	4 (4)	11 (14)	
Disease duration (years)	10.3 (9.2)	9.3 (9.0)	11.6 (9.3)	0.132^b^
EDSS score, median (range)	2.0 (0–6.5)	2.0 (0–6.5)	2.0 (0–6.5)	0.014^b^
Current use of DMT, *n* (%)	147 (81.2)	84 (84)	63 (77.8)	0.382^b^
zBRB	0.01 (0.71)	0.42 (0.48)	−0.51 (0.61)	<0.001^b^
HADS score, median [IQR]	9 (5–15)	8 (4–14)	10 (6–16)	0.125^b^
**Neuroimaging data**				
nLv (cm^3^)	9.43 (12.82)	6.32 (7.22)	13.27 (16.69)	<0.001^b^
nGMv (cm^3^)	782.35 (61.14)	794.43 (52.29)	767.44 (67.96)	0.003^c^
Nodal strength	11.68 (1.69)	12.140 (1.37)	11.110 (1.88)	<0.001^b^
Local efficiency	0.363 (0.02)	0.367 (0.02)	0.359 (0.03)	0.033^b^
Cluster coefficient	0.261 (0.02)	0.264 (0.01)	0.257 (0.02)	0.027^b^
Transitivity	0.245 (0.02)	0.249 (0.02)	0.241 (0.02)	0.007^b^
Global efficiency	0.289 (0.02)	0.295 (0.02)	0.282 (0.03)	0.003^b^
Assortativity	0.012 (0.03)	0.008 (0.03)	0.016 (0.03)	0.053^b^

*The data represent the absolute numbers and proportions of the qualitative data, and the mean and SD for the quantitative data, unless otherwise specified. IQR, interquartile range; PwMS-CP/CI, patients with multiple sclerosis cognitively preserved/cognitively impaired; CRQ, Cognitive Reserve Questionnaires; RRMS, relapsing remitting multiple sclerosis; SPMS, secondary progressive multiple sclerosis; EDSS, Expanded Disability Status Scale; DMT, disease-modifying treatment; zBRB, global cognitive performance score; HADS, Hospital Anxiety and Depression Scale; nLv, normalized lesion volume; nGMv, normalized gray matter volume*.

a *Chi-squared test*;

b *Wilcoxon–Mann–Whitney test*;

c *Student's t-test*.

### Relationship Between CR, Structural Connectivity, and Cognition

We found significant associations between CRQ and zBRB scores in the entire cohort (β = 0.324, 95% confidence interval, CI: 0.24–0.41, *p* < 0.001), in PwMS-CP (β = 0.253, 95% CI: 0.17–0.34, *p* < 0.001; Hedges' *g*: 0.521, 95% CI: 0.22–0.82), and also in PwMS-CI (β = 0.300, 95% CI: 0.19–0.41, *p* < 0.001; Hedges' *g*: 0.626, 95% CI: 0.33–0.93). In parallel, significant associations were also found between graph structural connectivity properties and cognitive scores in the entire cohort and in the PwMS-CI group in all studied graph measures except for assortativity (entire PwMS cohort β between 0.215 and 0.285, 95% CI: 0.12–0.38, *p* < 0.001) (Table 2).

**Table 2:** Associations between graph structural connectivity properties and cognition in both PwMS groups.

	**PwMS-CP (*****n*** **=** **100)**			**PwMS-CI (*****n*** **=** **81)**		
	**β (95% CI)**	**Hedges' *g* (95% CI)**	***p*-value**	**β (95% CI)**	**Hedges' *g* (95% CI)**	***p*-value**
Nodal strength	0.023 (−0.10 to 0.15)	0.046 (−0.25 to 0.34)	0.710	0.282 (0.18 to 0.38)	0.585 (0.29 to 0.89)	<0.001
Local efficiency	0.029 (−0.09 to 0.14)	0.058 (−0.24 to 0.35)	0.710	0.230 (0.13 to 0.33)	0.471 (0.17 to 0.77)	<0.001
Cluster coefficient	0.024 (−0.09 to 0.14)	0.048 (−0.25 to 0.34)	0.710	0.218 (0.11 to 0.32)	0.445 (0.15 to 0.74)	<0.001
Transitivity	0.030 (−0.08 to 0.15)	0.06 (−0.23 to 0.35)	0.710	0.225 (0.12 to 0.33)	0.460 (0.16 to 0.76)	<0.001
Global efficiency	0.032 (−0.09 to 0.15)	0.064 (−0.23 to 0.36)	0.710	0.268 (0.17 to 0.37)	0.554 (0.26 to 0.85)	<0.001
Assortativity	0.059 (−0.04 to 0.16)	0.118 (−0.18 to 0.41)	0.710	−0.101 (−0.23 to 0.03)	−0.202 (−0.5 to 0.09)	0.132

*Beta coefficients and 95% confidence intervals (CI) from age- and gender-adjusted linear regression models. PwMS-CP/CI, patients with multiple sclerosis cognitively preserved/cognitively impaired. P-values were adjusted by FDR*.

Regarding the relationship between the CRQ and structural connectivity, we found a very low effect size association in PwMS-CP and a low-to-moderate correlation in PwMS-CI. However, after multiple comparisons, those associations did not reach statistical significance (*p* < 0.05). Nodal strength, transitivity, and global efficiency were the metrics showing a moderate effect size in this group (Table 3).

**Table 3:** Associations between CRQ and graph structural connectivity properties in both PwMS groups.

	**PwMS-CP (*****n*** **=** **100)**			**PwMS-CI (*****n*** **=** **81)**		
	**β (95% CI)**	**Hedges' *g* (95% CI)**	***p*-value**	**β (95% CI)**	**Hedges' *g* (95% CI)**	***p*-value**
Nodal strength	−0.072 (−0.24 to 0.10)	−0.144 (−0.48 to 0.15)	0.490	0.267 (0.03 to 0.50)	0.552 (0.25 to 0.85)	0.068^*^
Local efficiency	−0.114 (−0.29 to 0.06)	−0.229 (−0.52 to 0.07)	0.412	0.200 (−0.05 to 0.45)	0.407 (0.11 to 0.70)	0.136
Cluster coefficient	−0.080 (−0.26 to 0.10)	−0.160 (−0.45 to 0.13)	0.490	0.187 (−0.06 to 0.43)	0.379 (0.08 to 0.67)	0.136
Transitivity	−0.056 (−0.24 to 0.13)	−0.112 (−0.41 to 0.18)	0.547	0.258 (0.02 to 0.50)	0.532 (0.23 to 0.83)	0.068^*^
Global efficiency	−0.113 (−0.28 to 0.06)	−0.226 (−0.52 to 0.07)	0.412	0.254 (0.01 to 0.50)	0.523 (0.23 to 0.82)	0.068^*^
Assortativity	0.172 (−0.03 to 0.37)	0.348 (0.05 to 0.64)	0.412	0.239 (0.02 to 0.46)	0.490 (0.19 to 0.79)	0.068^*^

*Beta coefficients and 95% confidence intervals (CI) from age- and gender-adjusted linear regression models. PwMS-CP/CI, patients with multiple sclerosis cognitively preserved/cognitively impaired. P-values were adjusted by FDR*.

* *P < 0.05 before correcting for multiple comparisons by FDR*.

### Models to Explain Cognitive Performance

**Figure 2:**
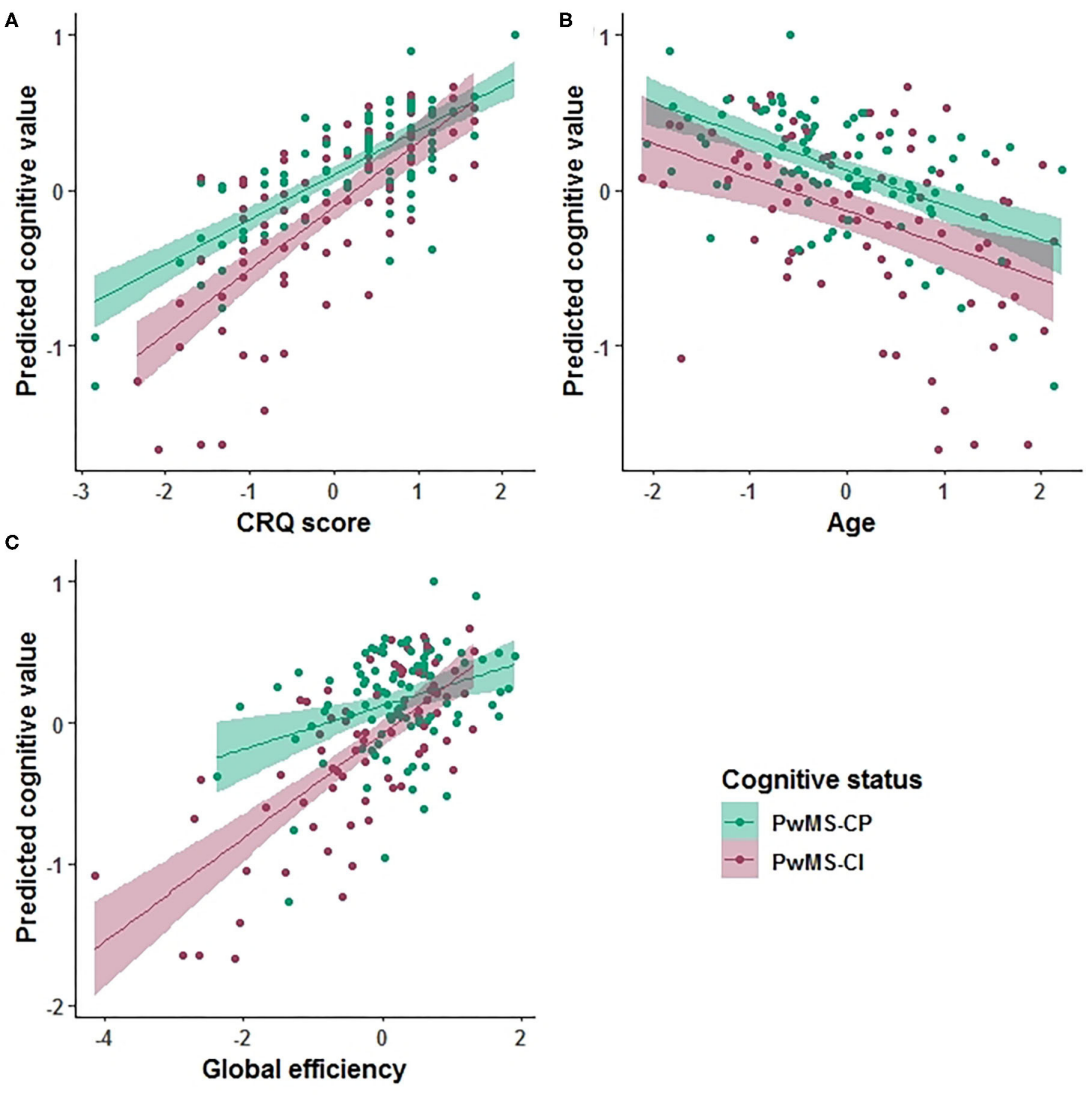
Prediction value in the cognitive explanatory model. Marginal effects of the Cognitive Reserve Questionnaire (CRQ) score **(A)**, age **(B)**, and global efficiency **(C)** are shown. The PwMS-CP group is colored green and the PwMS-CI is represented in red.

Based on the AIC, the final multiple linear regression model included CRQ score, age, gender, the HADS score, global and local efficiency, transitivity, and nLv as variables associated with cognitive performance. This model was applied to each group of patients separately. In PwMS-CP, 34% of the cognitive performance (mean zBRB score) was explained by the model (adj *R*^2^ = 0.34, *p* < 0.001). In this group, a one-point increase in the CRQ score was associated with a 0.26-point increase in the zBRB (the only significant variable in the model) (β = 0.259, 95% CI: 0.17–0.35, *p* < 0.001). The CRQ showed a moderate association with cognitive performance (Hedges' *g* = 0.534, 95% CI: 0.24–0.83). In the PwMS-CI, 55% of the variability in the zBRB was explained by the model (adj *R*^2^ = 0.55, *p* < 0.001). In these patients, a one-point increase in the CRQ score was associated with a 0.18-point increase in the zBRB (β = 0.179, 95% CI: 0.07–0.29, *p* = 0.012). Moreover, in this model age (β = −0.119, 95% CI: −0.21 to −0.02, *p* = 0.041) and global efficiency (β = 0.504, 95% CI: 0.18–0.83, *p* = 0.012) were significantly associated negatively and positively with the zBRB, respectively (Table 4 and Figure 2). Transitivity and global efficiency were the variables showing a moderate and high association with cognition (transitivity Hedges' *g* = −0.590, 95% CI: −0.89 to −0.29 and global efficiency Hedges' *g* = 1.162, 95% CI: 0.85–1.48; Table 4).

**Table 4:** Associations between clinical and MRI variables and cognitive performance in both PwMS groups.

**Parameters**	**PwMS-CP (*****n*** **=** **100)**			**PwMS-CI (*****n*** **=** **81)**		
	**β (95% CI)**	**Hedges' *g* (95% CI)**	***p*-value**	**β (95% CI)**	**Hedges' *g* (95% CI)**	***p*-value**
CRQ score	0.259 (0.17 to 0.35)	0.534 (0.24 to 0.83)	<0.001	0.179 (0.07 to 0.29)	0.362 (0.07 to 0.66)	0.012
Age	−0.097 (−0.20 to 0.00)	−0.194 (−0.49 to 0.10)	0.165	−0.119 (−0.21 to −0.02)	−0.239 (−0.53 to 0.06)	0.042
Gender	0.198 (−0.01 to 0.41)	0.402 (0.106 to 0.70)	0.165	0.179 (−0.03 to 0.39)	0.362 (0.07 to 0.66)	0.121
HADS score	−0.083 (−0.18 to 0.01)	−0.166 (−0.46 to 0.13)	0.165	−0.079 (−0.18 to 0.02)	−0.158 (−0.45 to 0.14)	0.141
Local efficiency	0.087 (−0.19 to 0.37)	0.174 (−0.12 to 0.48)	0.585	−0.084 (−0.38 to 0.21)	−0.168 (−0.46 to 0.13)	0.567
Transitivity	−0.156 (−0.42 to 0.11)	−0.315 (−0.61 to −0.02)	0.404	−0.284 (−0.56 to 0.01)	−0.590 (−0.89 to −0.29)	0.087
Global efficiency	0.165 (−0.17 to 0.50)	0.333 (0.04 to 0.63)	0.440	0.504 (0.18 to 0.83)	1.162 (0.85 to 1.48)	0.012
nLv (cm^3^)	0.047 (−0.12 to 0.22)	0.094 (−0.20 to 0.39)	0.585	−0.103 (−0.21 to 0.00)	−0.206 (−0.45 to 0.09)	0.094

*Beta coefficients and 95% confidence intervals (CI) from a multiple linear regression model. PwMS-CP/CI, patients with multiple sclerosis cognitively preserved/cognitively impaired; CRQ, Cognitive Reserve Questionnaires; HADS, Hospital Anxiety and Depression Scale; nLv, normalized lesion volume. P-values were adjusted by FDR*.

We evaluated the predictive value of CRQ and global efficiency on cognitive status. The age- and gender-adjusted association between CRQ score and global efficiency on having an impaired cognitive status was −0.338 (OR: 0.71, 95% CI: 0.52–0.97, *p* = 0.036) for CRQ score and −0.531 (OR: 0.59, 95% CI: 0.41–0.82, *p* = 0.002) for global efficiency.

## Discussion

In this study, we set out to understand the protective effect of CR on structural network integrity and their impact on cognition in PwMS in relation to other important demographic and MS-related factors. As such, we explored the relationship between CR and structural brain connectivity and analyzed different determinants of neuropsychological performance, including clinical information and some metrics of brain burden, in the presence or absence of CI. Although we found a marginal association between CR and structural connectivity integrity, it is only after brain damage reaches a significant level and CI is present that we found a moderate association between these measures. While the CR is the only variable associated with cognition in patients with good cognitive performance, when CI flourishes, structural brain damage, and aging are also related to this parameter. Indeed, in PwMS-CI, the impact of network integrity dysfunction is stronger than the effect of lifelong intellectual enhancement. The observed benefit of CR on cognitive performance has practical implications, including the implementation of strategies for intellectual life enrichment in addition to conventional therapies to palliate the effect of brain damage.

The relationship between CR and cognition has been studied in several neurological diseases, including MS (7). It has been suggested that more intellectual enrichment potentially protects PwMS from cognitive decline (7, 14, 35). Indeed, we found that higher scores in the CRQ scale were associated with better cognitive performance, meaning that CR could help to preserve the cognitive function. However, to the best of our knowledge, the effect that CR may have on structural connectivity networks remains unknown. In this regard, we observed a relationship between structural brain connectivity dysfunction and CRQ scores with low-to-moderate effect sizes. Specifically, in the PwMS-CI group, lower scores of CRQ were associated with decreased nodal strength, transitivity, and global efficiency. Thus, CR might have a positive effect on the integration mechanisms that support long-range connections (36) and on network segregation, reflecting compensatory mechanisms against cerebral damage. Other studies focusing on functional networks found links between CR and network efficiency in healthy elderly individuals (15) and in PwMS (12), which makes the protective role of CR more plausible on functional than on structural connectivity. Considered together, a higher CR tends to ameliorate the negative impact of MS on brain connectivity and seems to protect against cognitive decline.

Investigating the interaction between CR and structural connectivity on cognitive performance, we demonstrate the protective effect of intellectual life enrichment assessed with the CRQ on cognition in PwMS, with and without CI, irrespective of age, mood disorders, and brain burden. More specifically, we studied CR and structural connectivity integrity along with clinical and more conventional MRI parameters of brain damage in a model that explains cognitive performance in patients with different cognitive status. In the PwMS-CP group, CR explains the 34% of the variance in neuropsychological performance, whereas in the PwMS-CI group, CR together with age and global efficiency explain the 55% of the variance in cognitive performance. In this latter group, the association between CR and cognition was weaker than in the PwMS-CP cohort. The link between aging and structural brain connectivity with cognition in PwMS-CI was expected, since, as patients get older and brain damage due to pathological events accumulates, its impact on cognition augments. Aging is known to promote alterations in neuronal structure, loss of synapses, and dysfunction of neuronal networks (37). Also, previous studies have described a decreased global efficiency on PwMS compared to healthy volunteers, suggesting a disrupted topological organization of the WM networks due to impaired structural connections (38). Besides, abnormalities of global efficiency have been associated with negative consequences on cognition impacting different cognitive domains such as memory and attention performance (39–41). As the compensation and adaptation of brain mechanisms probably deteriorate with age and with brain damage, it would appear that brain network dysfunction leads to CI (16). Overall, our results reinforce the protective capacity of CR at any stage of the disease, including in PwMS that suffer cognitive decline.

Our findings entail relevant clinical repercussions as they emphasize the use of the CRQ scale in routine clinical practice to achieve a comprehensive assessment of PwMS and to identify at-risk individuals of cognitive decline. Neurologists should recommend that PwMS participate in early interventions to maximize their brain resources, such as intellectual enhancement or neuropsychological programs.

Our study is not absent of limitations, particularly as our cohort was composed predominantly of relapsing-remitting MS patients, and thus, it limits the capacity to generalize these findings to more advanced phenotypes. However, this is the most common phenotype encountered in the clinic in the current treatment era, with lower rates of worsening and evolution to SPMS in patients compared to earlier natural history cohorts (42). Furthermore, despite the fact that CRQ scores were different in the PwMS-CP and PwMS-CI groups, results remained unchanged when we balanced CRQ scores (data not shown). In addition, CR cannot be measured directly and there is still no consensus as to what is the best proxy for CR (43). Nevertheless, the CRQ measures different intellectual enrichment factors addressing experiences throughout life and is easily applicable in the clinical field due to its brevity and the absence of open responses (22). We do not have longitudinal data on cognitive performance so we were unable to establish a causal effect, yet our results are promising and in accordance with the existing literature. Finally, the inclusion of fMRI in future studies might be useful to further explore compensatory and plasticity mechanisms driven by intellectual enrichment in MS.

In conclusion, CR could have a positive effect on the connectivity of the brain network, which can be observed in patients with more severe clinical impairment. The results presented here highlight the important protective value of CR on cognitive performance, regardless of cognitive status. However, once CI has flourished, over and above the effect of CR, cognition is also influenced by the presence of structural brain damage and aging. This study draws attention to the benefits of promoting an intellectually rich lifestyle in PwMS, as it may have an important impact on their future cognitive status through all stages of the disease.

## Data Availability Statement

The raw data supporting the conclusions of this article will be made available by the authors, without undue reservation.

## Ethics Statement

The studies involving human participants were reviewed and approved by Ethical Committee at the Hospital Clinic Barcelona. The patients/participants provided their written informed consent to participate in this study.

## Author Contributions

All authors listed have made a substantial, direct and intellectual contribution to the work, and approved it for publication.

## Conflict of Interest

NS-V received compensation for consulting services and speaker honoraria from Genzyme-Sanofi, Almirall, Novartis, Merck, and Biogen. MS received speaker honoraria from Genzyme, Novartis, and Biogen. IP-V holds a patent for an affordable eye tracking system to measure eye movement in neurological diseases and holds stock options in Aura Innovative Robotics. YB received speaking honoraria from Biogen, Novartis, and Genzyme. EM-L received grants for the institution for research and educational purposes from Sanofi-Genzyme and Novartis. She received travel support for international and national meetings over the last 3 years from Roche and Sanofi-Genzyme. She has received honoraria for consultancies from Roche and Sanofi. She has been a member of the working committee of International Multiple Sclerosis Visual System (IMSVISUAL) Consortium. From 16 April 2019, she works in the European Medicine Agency (Division E Evaluation); AS received compensation for consulting services and speaker honoraria from Bayer-Schering, Merck-Serono, Biogen-Idec, Sanofi-Aventis, TEVA, Novartis, and Roche. SL received compensation for consulting services and speaker honoraria from Biogen Idec, Novartis, TEVA, Genzyme, Sanofi, and Merck. MA holds equities in Bionure and Goodgut. The remaining authors declare that the research was conducted in the absence of any commercial or financial relationships that could be construed as a potential conflict of interest.
