# Supplementary material for: Impact of Cognitive Reserve and Structural Connectivity on Cognitive Performance in Multiple Sclerosis
Source: Front Neurol. 2020 Oct 30;11:581700. doi: 10.3389/fneur.2020.581700 (PMC7662554; doi:10.3389/fneur.2020.581700)
Supplement: Supplementary file 1 [file Data_Sheet_1.PDF]

**Supplementary material**

MRI acquisition and processing

The acquisition parameters for the 3D-structural MPRAGE were: repetition time (TR) = 1800 ms; echo time (TE) = 3.01 ms; inversion time (TI) = 900 ms; 240 sagittal slices with 0.94 mm isotropic voxel size and 256x256 matrix size. The 3DT2 FLAIR sequence parameters were: TR = 5000 ms; TE = 304 ms; TI = 1800 ms; 192 sagittal slices with 0.94 mm isotropic voxel size and 6256 x 256 matrix size. Finally, High Angular Resolution Diffusion Imaging (HARDI) data was acquired with: TR = 14800 ms; TE = 103 ms; 100 contiguous axial slices; 1.5 mm isotropic voxel size; 154x154 matrix size; b value = 1000 s/$mm^2$; 60 diffusion encoding directions and a single baseline image acquired at 0 s/$mm^2$. Field map images were also generated to correct the distortions caused by field in homogeneities (echo time 1/echo time 2 = 4.92/7.38 ms, with the same slice prescription, slice thickness and field of view as the HARDI sequence).
